# Observation‐Only Management of Cervicofacial Nontuberculous Mycobacterial Lymphadenitis in Children

**DOI:** 10.1002/lary.70029

**Published:** 2025-08-11

**Authors:** Cheyenne Roohani, Amanda S. Evans, Stephen R. Chorney, Christopher C. Liu

**Affiliations:** ^1^ Department of Otolaryngology—Head & Neck Surgery University of Texas Southwestern Medical Center Dallas Texas USA; ^2^ Children's Health Division of Pediatric Otolaryngology Dallas Texas USA; ^3^ Department of Pediatrics—Infectious Disease University of Texas Southwestern Medical Center Dallas Texas USA

**Keywords:** atypical mycobacteria, cervicofacial lymphadenitis, nontuberculous mycobacteria, observation

## Abstract

**Objectives:**

To determine the natural history of nontuberculous mycobacterial (NTM) lymphadenitis in immunocompetent children managed nonoperatively.

**Methods:**

A retrospective study including patients (< 18 years old) diagnosed with cervicofacial NTM lymphadenitis at a tertiary children's hospital between 2017 and 2023. Patients were either observed or surgically treated. Analysis focused on observation outcomes. Surgical patients were included for comparative context.

**Results:**

Seventy‐two patients were included; 45 (63%) were managed by observation‐only and 27 (37%) underwent surgical excision. Baseline characteristics were similar between groups. Median follow‐up was longer in the observation group (123 days [IQR: 92–264]) than in the surgical group (83 days [IQR: 60–98], *p* < 0.001). Among observed patients, median time to resolution was 168.5 days (IQR: 97–291). Kaplan–Meier‐estimated cumulative resolution was 26.3% at 4 months, 42.7% at 6 months, and 82.8% at 12 months. Older age predicted faster resolution (HR: 1.22, 95% CI: 1.04–1.42, *p* = 0.01), while parotid involvement predicted slower resolution (HR: 0.21, 95% CI: 0.06–0.72, *p* = 0.01). One patient in the observation group developed a keloid requiring scar revision. In the surgical group, three patients (11%) experienced marginal mandibular nerve weakness.

**Conclusions:**

Observation‐only management of cervicofacial NTM lymphadenitis in children resulted in high rates of spontaneous resolution by 12 months with minimal morbidity. While surgical excision provided faster resolution, it carried a higher risk of facial nerve injury. Observation may be a safe, effective alternative for selected patients, potentially avoiding surgical complications.

**Level of Evidence:**

3.

## Graphical abstract

This is a retrospective study of immunocompetent children with nontuberculous mycobacterial cervicofacial lymphadenitis who were managed by observation. Kaplan–Meier‐estimated cumulative resolution was 82.8% by 12 months. Observation may be a safe, effective alternative to surgical excision for selected patients.

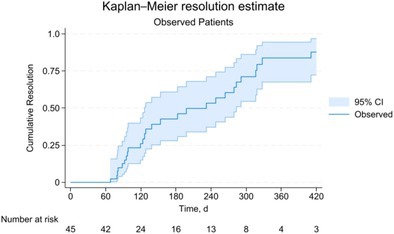

## 1 Introduction

Nontuberculous mycobacteria (NTM) constitute a diverse group of mycobacteria inhabiting various ecological niches that present a clinical challenge given their ubiquity and variable pathogenicity. In immunocompetent children under the age of 5, these organisms primarily manifest as chronic cervicofacial lymphadenitis [1, 2]. Clinical presentation typically involves a slow‐growing, non‐tender mass without constitutional symptoms, potentially progressing to violaceous discoloration and sinus tract formation. The mode of transmission is presumed to be oral exposure to contaminated sources such as soil, water, food, or aerosols, possibly explaining the predilection for the cervicofacial region in this age group [3]. Although the global incidence of mycobacterial infections in children varies, ranging from 0.8 to 4.5 cases per 100,000 individuals annually across different age brackets [4, 5], there is a noted upward trend, suggesting a growing burden for healthcare providers managing pediatric cases [6, 7].

Traditionally, surgical excision has been the primary treatment recommendation for NTM infections, aiming for complete removal of affected lymph nodes and surrounding tissue [3, 8, 9, 10, 11]. Alternative surgical interventions like incision and drainage or curettage have fallen out of favor due to inferior outcomes and concern for fistulization [9, 12, 13, 14, 15]. Although complete excision demonstrates efficacy with low recurrence rates, it carries a risk of transient or permanent facial nerve paralysis, occurring in 10%–25% and 2%–4% of cases, respectively, depending on location [16, 17, 18]. The close proximity to the facial nerve also affects how completely the disease can be excised. Despite these concerns, surgery remains the cornerstone of treatment.

Observation‐only management has received relatively little attention, with few studies examining this approach [19, 20, 21]. Given the low but concerning surgical risk, further investigation into the safety and efficacy of nonoperative management is warranted. This study aims to address this gap by characterizing the clinical course, resolution rates, and potential risks associated with observation‐only management of cervicofacial NTM lymphadenitis in children. We hypothesize that observation is a viable alternative to surgery in select cases, with an acceptably high rate of spontaneous resolution and minimal risk of complications. By doing so, we hope to inform clinical decision‐making and support a more individualized approach to the management of this increasingly common pediatric condition.

## 2 Methods

### 2.1 Data Collection

We conducted a retrospective review of children (age < 18 years) diagnosed with cervicofacial NTM lymphadenitis in the pediatric otolaryngology clinic at Children's Medical Center in Dallas, TX, and Plano, TX, over an 8‐year period (2016–2023). Review and dissemination of this data at Children's Medical Center was approved by the University of Texas Southwestern Institutional Review Board (IRB# 2019‐1666). Patients were identified using International Classification of Diseases 10th revision (ICD‐10) codes A31.1 (cutaneous mycobacterial infection), A31.8 (other mycobacterial infections), and A31.9 (mycobacterial infection, unspecified). Patients' charts were then manually reviewed to confirm the diagnosis of cervicofacial NTM lymphadenitis. Patients were excluded if they did not have at least one follow‐up appointment or had NTM infections outside the head or neck. In concordance with the 2023 consensus guidelines on the diagnosis and management of NTM infections, definite NTM lymphadenitis was defined by positive mycobacterial culture or PCR results, while probable cases were based on characteristic clinical presentation and concordant investigations such as imaging findings [22]. Clinical findings included painless, unilateral lymphadenopathy with or without violaceous skin changes and the absence of constitutional symptoms such as fever or malaise. Demographic data that was collected included patient age, sex (male or female), race (White, Black or African American, Native Hawaiian or Other Pacific Islander, Asian, and American Indian or Alaska Native), and ethnicity (Hispanic or Latino, not Hispanic or Latino). Clinical data collected included location of infection, dates of infection and resolution, surgery date (if applicable), imaging, laboratory values, medical comorbidities, long‐term antibiotics (> 4‐week course), and complications by manually reviewing inpatient, clinic, and operative notes.

### 2.2 Counseling

All patients were evaluated by one of seven attending surgeons from the Division of Pediatric Otolaryngology at Children's Health. Caregivers were given information on management options including surgery as well as observation‐only. Risks of surgery including the risk of injury to the facial nerve or its branches (when relevant) are discussed with families. Similarly, the time course, risk of scarring, and the likelihood of fistulization are discussed regarding observation‐only management. Using the information provided regarding the risks and benefits of either option, the caregiver made the final decision regarding treatment. Caregivers were also informed that they could opt for surgery at any time during observation. Antibiotics are not routinely offered as a treatment option.

### 2.3 Data Analysis

The data were collected and stored in a REDCap database. The date that the caregivers first noticed any swelling or abnormality was the date of symptom onset and was extracted from clinic and emergency department notes. The date of resolution was the date that a provider documented complete resolution of disease. Resolution was determined by cessation of drainage, healing of the sinus tract, no further palpable mass, and resolution of overlying skin erythema. Patients in whom the date of resolution was not able to be determined either due to being lost to follow‐up or not having yet resolved by the study end date were censored.

The primary outcome measure was time to infection resolution within 12 months, analyzed using Kaplan–Meier time‐to‐event analysis. Kaplan–Meier curves were used to provide a graphical representation of the probability of resolution over time for both the observation and surgical cohorts, accounting for censored data, and to visualize the time course of resolution rates. However, the surgical group was used as a reference point for expected resolution and complications, rather than as a control. Based on local practice patterns and previous literature demonstrating time to resolution of over 15 months [21] we focused on a 12‐month timeframe to estimate resolution and allow adequate time for children to heal. The estimated cumulative incidence of resolution at 4, 6, and 12 months was calculated. Surgical complications, scarring requiring revision surgery, and failure of observation‐only management (conversion from observation to surgical management) were secondary outcome measures. Continuous data were presented as medians with interquartile ranges (IQR) while categorical data were presented as counts with percentages. Comparison between groups was performed using Wilcoxon rank‐sum (Mann–Whitney) for continuous data and Fisher's exact test for categorical data. Cox proportional hazard ratios were calculated along with log‐rank testing for comparison of time‐to‐event curves. All statistics were performed with Stata (StataCorp, 2021. Stata Statistical Software: Release 17. College Station, TX: StataCorp LLC).

## 3 Results

A total of 72 patients were included in the study, with 45 patients (63%) managed by observation‐only. Table 1 compares baseline characteristics between children managed with observation and those managed with surgery (*N* = 27). There were no differences in patient characteristics between groups. Median length of follow‐up was longer in the observation group (123 days [IQR: 92–264] vs. 83 days [IQR: 60–98], *p* < 0.001). The only complication observed in the observation group was one patient that developed a keloid around the scar, for which they underwent scar revision surgery.

**TABLE 1:** Baseline characteristics of observed vs. surgical cervicofacial NTM.

Characteristic	Observation group (*N* = 45)	Surgical group (*N* = 27)	*p*
Median age, years (IQR)	2.0 (1.0–5.0)	2.0 (1.0–3.0)	0.30
Male, *n* (%)	17 (38)	7 (26)	0.44
Median follow up, day (IQR)	123 (92–264)	83 (60–98)	< 0.001
Race, *n* (%)			
White	32 (71)	21 (78)	0.97
Black or African American	5 (11)	3 (11)	
Asian	1 (2.2)	0	
Other	7 (15)	3 (11)	
Hispanic ethnicity, *n* (%)	24 (53)	13 (48)	0.81
Definitive NTM, *n* (%)	9 (20)	9 (33)	0.26
Location, *n* (%)			
Parotid	8 (18)	5 (19)	0.99
Facial	3 (6.7)	1 (3.7)	0.99
Submandibular	21 (47)	10 (37)	0.47
Cervical level 2	11 (24)	11 (41)	0.19
Cervical level 3	2 (4.4)	1 (3.7)	0.99
Cervical level 4	1 (2.2)	0	0.99
Cervical level 5	3 (6.7)	3 (11)	0.67
Antibiotic therapy > 4 weeks, *n* (%)	2 (4.4)	3 (11)	0.36
BMI percentile median (IQR)	63 (35–93)	62 (42–85)	0.98

Among the surgical cohort, three (11%) of the patients had marginal mandibular nerve injury; but due to limited follow up, it is not known if these were transient or permanent injuries. The mean time of follow up after surgery was 20 days for these patients. Two cases involved the submandibular region, and one involved the parotid extending into level II.

**FIGURE 1:**
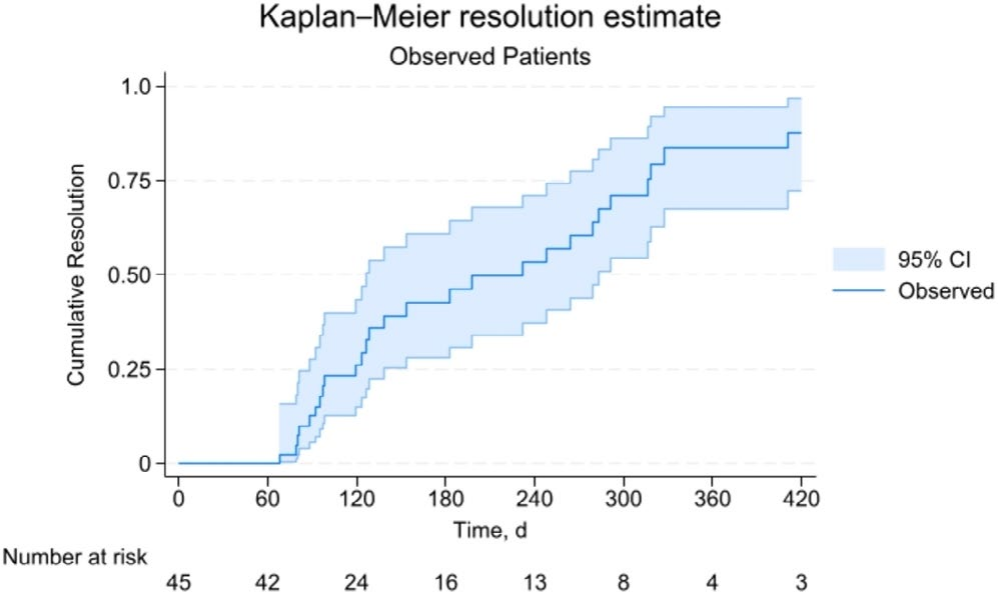
Kaplain–Meier failure curve for observed patients with 95% CI.

**FIGURE 2:**
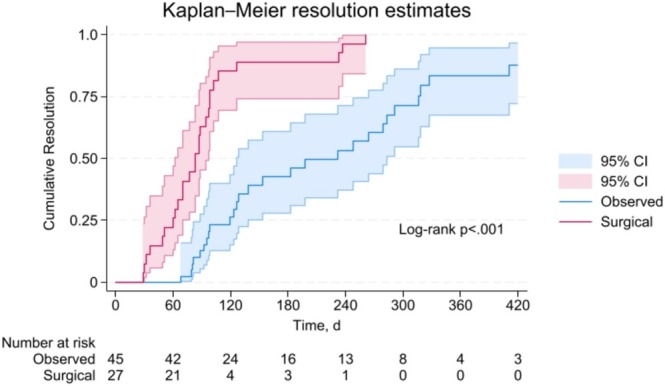
Kaplain–Meier failure curves for observed and surgical patients with 95% CI.

**FIGURE 3:**
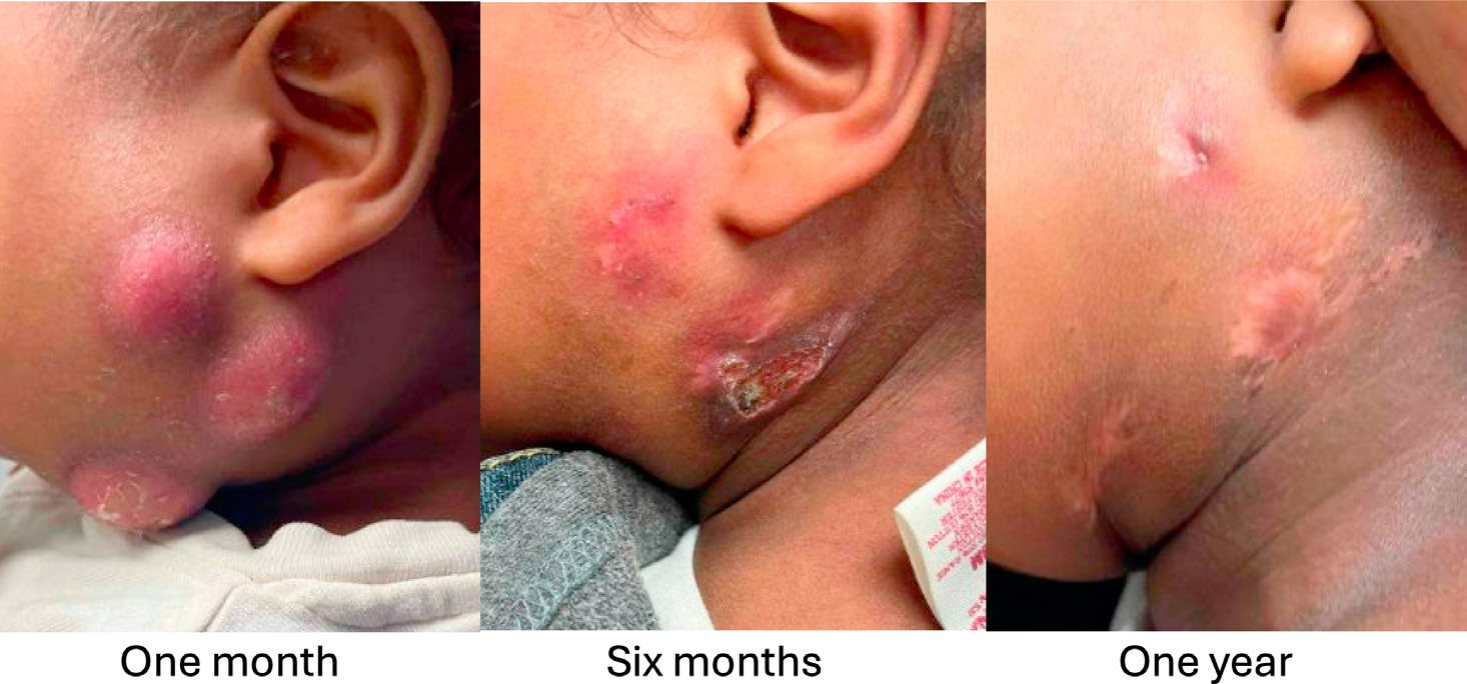
Photographs of a patient with multi‐focal disease who was observed.

In patients being observed, cumulative resolution estimates were 26.3% (95% CI: 15.1–43.4) at 4 months, 42.7% (95% CI: 28.1–60.9) at 6 months, and 82.8% (95% CI: 67.0–93.9) at 12 months. The Kaplan–Meier resolution estimates with 95% CI are shown in Figure 1. Censoring occurred due to children being lost to follow‐up or lacking documented resolution by the study end date. The Cox proportional hazards model predicting time to resolution in the observation group is shown in Table 2. Older age was associated with faster times to resolution (HR: 1.22, 95% CI: 1.04–1.42, *p* = 0.01) while parotid involvement was associated with a slower time to resolve (HR: 0.21, 95% CI: 0.06–0.72, *p* = 0.01). Figure 2 compares time to an event for observation and surgical groups for comparison. For surgically managed children in this series, cumulative resolution via surgery at 4 months was 85.2% (95% CI: 69.6–95.3). There were differences in time to resolution curves between the observation only and surgery excision groups as demonstrated by a log‐rank test (*p* < 0.001). Figure 3 is a photographic documentation of a patient whose caregivers elected observation.

**TABLE 2:** Cox proportional hazards model predicting time to resolution (observation group).

Variable	HR	Std. err	*z*	*p*	95% CI
Age	1.22	0.10	2.45	0.01	1.04–1.42
Parotid	0.21	0.13	−2.49	0.01	0.06–0.72
Definitive	0.35	0.19	−1.91	0.06	0.12–1.03

## 4 Discussion

This study underscores the safety and efficacy of observation‐based therapy for NTM lymphadenitis, with the majority of patients achieving resolution within 12 months. Unlike previous studies comparing surgical and nonsurgical approaches, our focus was solely on demonstrating the viability of observation‐only without significant morbidity. Caregivers were given the option of surgical excision versus observation. We acknowledge that certain factors may influence caregiver decision‐making, such as surgical risk associated with anatomic location, presence of skin involvement and fistulization, multi‐focal disease, recurrent disease, and caregiver concern for possible malignancy. Fistulization, a common occurrence in the natural history of NTM lymphadenitis, was not considered a complication in this study. Instead, caregivers were counseled that fistulization should be expected, and they were taught how to care for the wound should it fistulize. Patients who elected observation were seen monthly for the first 3 months. Depending on caregiver comfort, interval follow‐up appointments were then extended to every 3 months until resolution. No additional imaging was obtained during the observation period. None of the patients who were observed required a conversion to surgery for a “treatment failure.” In our study, “resolution” was defined as cessation of drainage, healing of the sinus tract, and absence of residual mass or skin changes—not necessarily cosmetic normalization. Importantly, caregivers' satisfaction with this endpoint was high, and scar‐related concerns were rare.

Antibiotic therapy as a first‐line treatment has been explored previously but lacks strong evidence for altering disease progression, often accompanied by significant side effects such as fever, gastrointestinal disturbance, fatigue, and tooth discoloration [4, 8, 11, 16, 21, 22, 23, 24, 25, 26, 27]. Given these findings, our institution does not routinely offer antibiotic therapy as a treatment option.

There are only two studies in the current literature that evaluate observation‐only management of NTM lymphadenitis. These studies support our findings, indicating high resolution rates with observation and minimal complications [19, 21]. In 2008, Zeharia et al. studied 92 children, all of whom were managed by observation alone, and found that 71% of infections had resolved by 6 months and the remainder by 12 months [19]. They had no serious complications in their cohort. Lindeboom compared observation to antibiotic management and found no significant difference between the two groups [21]. In this study, infections in the observation‐only group resolved within 16 months with a median of about 9 months, and the authors did not report any complications in their observation‐only cohort. Our study reports Kaplan–Meier‐derived resolution estimates for observation‐only management of NTM lymphadenitis, providing a time‐bound framework for counseling families. This can provide novel insights into patient‐centered outcomes such as scar satisfaction and caregiver decision‐making.

A commonly taught risk of observation is unfavorable scarring. In our experience, scar outcomes were favorable—only one family desired a scar revision surgery in a patient that developed a keloid. Additionally, we would argue that if the NTM infection has progressed to involving the skin (presence of violaceous discoloration), the size of the incision and defect created to resect affected skin is far longer than what would be required for an elective scar revision subsequent to the resolution of the infection. Furthermore, in cases where the infection is adjacent to branches of the facial nerve (submandibular, parotid, facial), operating poses a risk of permanent injury to one or more of these branches. For many families, this risk of facial nerve injury is not acceptable. This risk could be avoided by allowing the infection to resolve spontaneously and returning for scar revision surgery in an elective setting. The plane of dissection for a scar revision is much more superficial and would not place the facial nerve in jeopardy. We routinely offer scar revision surgery, but caregivers very infrequently request to proceed.

The main challenge with observation‐only management lies in caregivers coping with wound drainage. With proper counseling, this burden is manageable, and most families opt for observation over surgery, especially when facial nerve involvement is a concern. Our approach to counseling families and caregivers is to set expectations for the expected course of the disease early on. At the first visit, we educate the families to prepare for changes in color to the overlying skin, growth of the mass, and likely eventual drainage. We educate the families on management of drainage, which includes simple interventions such as using gauze and tape over the drainage site until the drainage subsides. Families are made aware that the wound may drain for weeks and that the entire process from initial presentation to resolution can take over a year. They are also made aware that at any point during the process we can re‐evaluate and change the management plan (including converting to operative management) should they feel that it is necessary. We have found that with early counseling and expectation setting at the first visit, families are prepared and feel comfortable caring for the wound and have not requested conversion to surgery in our experience.

There are several limitations to our study. Our sample size was small and further lessened by patients who had to be censored. Only a minority of our patients had definite NTM infections, meaning that most of our patients were diagnosed with probable NTM lymphadenitis. In order to be captured by our search, patients had to have at least one encounter in the pediatric otolaryngology clinic in our institution. Given our institution's practice and referral patterns, we have a high level of confidence that this captured most, if not all, patients who were diagnosed with NTM lymphadenitis. However, we must acknowledge that there may be patients with NTM lymphadenitis who were not referred to our clinic and thus were not able to be captured for this study.

Our study is a retrospective review of one treatment modality which was not in comparison to other treatment modalities such as surgery. The direct comparison of the Kaplan–Meier analysis between the observation and the surgical groups has its shortcomings. The time to recovery for the surgical patients is artificially influenced by factors such as operating room, surgeon, and family availability and is not a representation of the actual time to recovery following surgery. The shorter time to resolution in the surgical group should be interpreted cautiously, as this metric reflects logistical timing of surgery rather than time to physiologic healing. In contrast, our observation data capture the natural course of disease resolution and may be more representative of biologic behavior in the absence of intervention. The length of observation in this study may not be long enough to capture patients who recur due to sequestered or dormant infection.

A randomized controlled trial comparing observation to surgical excision, which considered quality of life metrics, scar appearance, perceived healing quality, number of phone calls or visits to the office, drainage and wound concerns, and cost, could all be used as metrics for a future study and would add greatly to this very limited fund of knowledge. Additionally, future studies on patient factors that could identify which patients would benefit from surgery versus observation are warranted. While we did not formally measure quality‐of‐life outcomes, anecdotal feedback during follow‐up visits suggested that most caregivers were comfortable managing drainage at home and expressed minimal distress related to the prolonged natural course of resolution. Future prospective studies should include caregiver‐reported outcomes to better assess the psychosocial impact of observation‐based management.

## 5 Conclusion

This study demonstrates that observation‐only management of cervicofacial NTM lymphadenitis in immunocompetent children can lead to spontaneous resolution in most cases within 1 year and with minimal associated morbidity. While surgical excision remains a reliable and expedient option for resolution, it carries a measurable risk of complications, including facial nerve injury. Our findings suggest that, in appropriately selected patients, a nonoperative approach is a safe and effective alternative in appropriately selected children that avoids the potential risks of surgery. These results support the inclusion of observation in the shared decision‐making process when counseling families and highlight the need for further prospective studies to refine patient selection and optimize individualized care strategies. This approach allows clinicians to tailor care based on family preference, disease location, and anticipated surgical risk.

## Conflicts of Interest

Stephen R. Chorney is an educational consultant for Smith & Nephew. The other authors declare no conflicts of interest.

## Data Availability

The data that support the findings of this study are available on request from the corresponding author. The data are not publicly available due to privacy or ethical restrictions.
